# Neuromelanins of Human Brain Have Soluble and Insoluble Components with Dolichols Attached to the Melanic Structure

**DOI:** 10.1371/journal.pone.0048490

**Published:** 2012-11-05

**Authors:** Mireille Engelen, Renzo Vanna, Chiara Bellei, Fabio A. Zucca, Kazumasa Wakamatsu, Enrico Monzani, Shosuke Ito, Luigi Casella, Luigi Zecca

**Affiliations:** 1 Institute of Biomedical Technologies, National Research Council of Italy, Segrate, Milan, Italy; 2 Department of Chemistry, Fujita Health University, School of Health Sciences, Toyoake, Aichi, Japan; 3 Department of General Chemistry, University of Pavia, Pavia, Italy; Virginia Commonwealth University, United States of America

## Abstract

Neuromelanins (NMs) are neuronal pigments of melanic-lipidic type which accumulate during aging. They are involved in protective and degenerative mechanisms depending on the cellular context, however their structures are still poorly understood. NMs from nine human brain areas were analyzed in detail. Elemental analysis led to identification of three types of NM, while infrared spectroscopy showed that NMs from neurons of substantia nigra and locus coeruleus, which selectively degenerate in Parkinson’s disease, have similar structure but different from NMs from brain regions not targeted by the disease. Synthetic melanins containing Fe and bovine serum albumin were prepared to model the natural product and help clarifying the structure of NMs. Extensive nuclear magnetic resonance spectroscopy studies showed the presence of dolichols both in the soluble and insoluble parts of NM. Diffusion measurements demonstrated that the dimethyl sulfoxide soluble components consist of oligomeric precursors with MWs in the range 1.4–52 kDa, while the insoluble part contains polymers of larger size but with a similar composition. These data suggest that the selective vulnerability of neurons of substantia nigra and locus coeruleus in Parkinson’s disease might depend on the structure of the pigment. Moreover, they allow to propose a pathway for NM biosynthesis in human brain.

## Introduction

**Figure 1:**
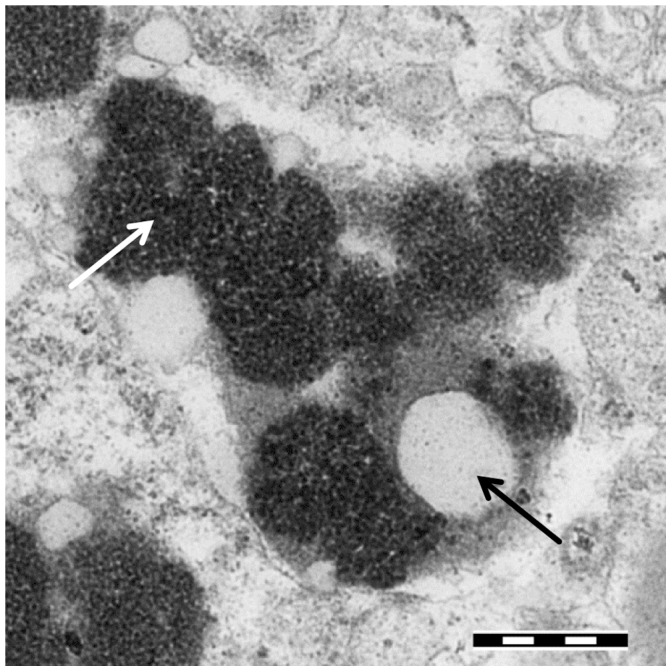
Transmission electron microscopy image of NM-containing organelles. Transmission electron microscopy image of a pigmented organelle from SN containing the NM pigment (white arrow) and lipid droplets (black arrow). The tissue was prepared as described in ref. 2 (scale bar 500 nm).

Neuromelanins (NMs) are a special class of compounds occurring in the brain of humans and animals that share with the better characterized melanins several structural, physical, and functional properties [1]. NM is contained in organelles together with lipid droplets (Figure 1). The abundance of NMs is quite variable in different animal species. NM accumulates with age and a higher content is found in long-living species like humans and primates, whereas only a very low content is found in common laboratory animals. Only recently it was shown that in humans NMs are present in all major brain regions, while in the past it was thought that only catecholaminergic neurons could accumulate NM [2]. It was also demonstrated that formation of NM is a protective process, as it removes cytotoxic quinones from the cytosol [3]. In addition, NM is able to block the toxicity of metal ions (Pb, Hg, Cd and others) by forming stable complexes, confirming the protective role of the pigment. The ability to chelate metal ions was attributed to the catechol groups of the melanic component. A major point of interest to investigate NM structure and function is related to the fact that in Parkinson’s disease (PD) the dopaminergic neurons containing NM selectively degenerate, while those without NM are spared from the neurodegenerative process [4]. Structural investigations demonstrated that poly-isoprenic lipids are a major component of NM, in addition to melanic and protein components [5]. However, important aspects of the NM structure are still unknown.

1. An open question is whether the melanic component of NM is similar to that present in synthetic and natural melanins. In fact, the amount of melanic component in NM appears to be lower than it would be expected on the basis of the high sulphur content.
2. The type of bond between the poly-isoprenic chain and the melanic component has not been identified.
3. The origin and structure of the protein component are unknown.
4. The arrangement of the above mentioned components remains to be elucidated.
5. X-ray powder diffraction measurements demonstrated an intriguing 4.7 Å structural motif, the origin of which is unknown [2].

One of the major difficulties concerning the elucidation of NM structure is the scarcity of biological sample. Concentration of NM in brain depends largely on the brain area, with substantia nigra (SN) and locus coeruleus (LC) containing the highest NM amounts, and on the age of the subject, as NM accumulates with age. NM can reach concentrations of 1.5–2.6 µg/mg tissue in most major brain regions of elderly subjects and up to 3.7 µg/mg tissue in SN [4]. Limitations in the access to the biological material are indeed stringent, considering that to obtain ∼1.0 mg of purified SN pigment, 4–5 human brains of suitable subjects are needed. This number rises to 30 subjects to obtain ∼0.5 mg of NM from LC tissues. It is therefore difficult to obtain even modest quantities of NM, and this shows the limitations in the type of analyses that can be performed on this particular biological substance. Another difficulty is the poor solubility of the pigment in non-destructive solvents. The part that can be solubilized in aqueous or organic solvents might not be representative for the entire pigment, which further complicates the interpretation of analytical and spectral data from techniques that only give information on the dissolved fraction. In this paper, a detailed analytical and spectroscopic investigation is reported on both the soluble and insoluble fractions of NM from various brain regions, with the aim of obtaining a more complete picture of the pigment structure. Until recently, only the NM from SN has been extensively studied. Comparison of SN-NM with the NMs of other brain regions might lead to new insights on the formation of the pigment in relation to the brain region of origin. For the first time, also NM from LC was studied in detail, which, despite its importance in neurodegenerative diseases, until now has received little attention because of the difficulty in obtaining the pigment in reasonable amounts. Understanding the structural details of NM could provide information regarding its biosynthetic pathway, e.g. the type of enzymes involved, the sulphur-containing molecules that are incorporated, the type of oligomeric precursors, etc. These aspects could help to clarify the site where each step of the formation of the pigment takes place, i.e. the cytosol, Golgi system, autophagic vacuoles, or lysosomes. These processes might show how the synthesis and accumulation of NM in dopamine (DA) containing neurons plays a protective role in neuronal viability.

## Materials and Methods

This study was approved by the Institutional Review Board of the Institute of Biomedical Technologies - National Research Council of Italy (Segrate, Milan, Italy) and was carried out in agreement with the Policy of National Research Council of Italy. Written informed consents for using brain samples for research purposes were obtained from closest relatives and are stored at the Section of Legal Medicine and Insurances, Department of Human Morphology and Biomedical Sciences, University of Milan. Final approval was given by the pathologist executing the autopsy. All tissues samples were analyzed anonymously.

### Isolation of the Pigment [2]

All samples of putamen (PUT), premotor cortex (CAX), cerebellum (CAB), globus pallidus (PAL), nucleus caudatus (CAU), corpus callosum (CAL), substantia alba or white matter from premotor cortex (SAB), SN and LC from human brain were obtained during autopsies of subjects without evidence of neuropsychiatric and neurodegenerative disorders. To obtain ∼1.5 mg NM, samples from each brain region were weighed (ranging from ∼1.0 g for SN to ∼20.0 g for CAL), and introduced into a 130 mL plastic centrifuge tube. Duration and volumes of incubation were determined based on total tissue weight, for ∼3.0 g tissue the following protocol was used: after grinding, 90 mL of water was added and the mixture was shaken. Tubes were centrifuged at 18,000 *g* for 15 min, and the pellet was washed twice with 90 mL of phosphate buffer (50 mM, pH 7.4). The sample was then incubated for 3 h at 37°C with 60 mL of Tris buffer (50 mM, pH 7.4) containing SDS (5 mg/mL). This suspension was centrifuged at 18,000 *g* for 20 min, the supernatant was removed, and the pellet was incubated for 3 h at 37°C with 20 mL of the same solution containing 0.2–0.5 mg/mL proteinase K (Sigma–Aldrich). The pigment was separated by centrifugation as above, washed twice with 5 mL of NaCl solution (9 mg/mL), and then with 3 mL of water. The sample was suspended in 2 mL of methanol and centrifuged, and the supernatant solution was removed. The sample was resuspended in 1 mL of hexane and centrifuged, and after eliminating the supernatant solution, the pigment was dried under nitrogen flow and placed under vacuum for 14 h at r.t. The organic fractions were combined and also dried under nitrogen flow.

### Determination of Amino Acid Content in Pigments

Isolated pigments (50 µg) were heated in 6.0 M HCl at 110°C for 21 h in an evacuated sealed glass tube [6]. The hydrolysate was dissolved in 100 µL of a pH 2.2 buffer for amino acid analysis. An aliquot of 80 µL (equivalent to 40 µg of pigment) was injected into a Hitachi model I-8500 amino acid analyzer. As the cysteine (and cystine) value can not be assessed under the above conditions, it was determined in all samples after HI hydrolysis as previously described [7].

### Elemental Analysis

Elemental analysis (C, H, N, S, O %) of NMs of the various brain regions (average of 2–9 samples per brain area) was carried out using the combustion technique as previously reported [8].

### Infrared Spectroscopy (IR)

IR spectra were obtained with a Perkin-Elmer Model 1600 FTIR spectrophotometer operating in the wavelength range 4000–400 cm^−1^. Before preparation of the samples, the specimens were stored in a dry box in the presence of P_2_O_5_ for 48 h to assure the same hydration degree in all of them. The samples were prepared as pellets (diameter 13 mm and thickness ∼0.20 mm) of NM (∼1 mg) in carefully dried potassium bromide (150 mg). The weight ratio between sample and KBr was chosen to avoid saturation effects in the absorption bands. For each sample 256 scans were collected and background noise was eliminated by subtraction of the spectrum of a KBr pellet acquired under the same conditions. To confirm the assignment of the various peaks to the different components, IR spectra were also registered on samples of synthetic melanin (SM) obtained by autoxidation of DA in the presence of cysteine, SM containing either 5% Fe (mol/mol DA) or 10% (w/w) bovine serum albumin (BSA; unpublished data), and on the lipids extracted from NM during the methanol and hexane washing steps. The IR spectrum of the lipid extract was registered after evaporation of a concentrated solution in CHCl_3_ between NaCl plates.

### Nuclear Magnetic Resonance (NMR) Spectroscopy

Solution NMR spectra were recorded on a Bruker AVANCE 400 spectrometer operating at a ^1^H frequency of 400.13 MHz at 293 K, using a z-grad z8202/184 ^1^H-^13^C inverse probe. Samples for NMR spectroscopy were prepared by stirring the pigment, typically 1.0 mg, very slowly in 0.7 mL of 99.8% dimethyl sulfoxide-*d_6_* (DMSO-*d*_6_) for 48 h. The undissolved material was removed by centrifugation before recording the spectra. The 1D proton NMR spectra were recorded using the standard Bruker microprogram *p3919gp* that applies the WATERGATE pulse sequence (water suppression by gradient-tailored excitation [9], [10]), generally using a delay for binomial water suppression of 150 µs and collecting 8192 scans. Line broadening (1.5 Hz) was used to increase the signal-to-noise ratio. Several types of gradient 2D NMR experiments were used to determine ^1^H-^1^H and ^1^H-^13^C connectivities. These included gradient correlation spectroscopy (DQF-COSY) for the determination of scalar ^1^H-^1^H connectivities, and heteronuclear single quantum correlation (HSQC) and heteronuclear multiple bond correlation (HMBC) for the determination of one-, two-, three-, and four-bond ^1^H-^13^C connectivities. Delays of 50 and 100 ms were employed to observe multiple-bond correlations.

Diffusion measurements in solution were performed using the standard Bruker microprogram *stebpgp1s19* to obtain diffusion ordered spectroscopy (DOSY) spectra at 293 K [11], [12]. The pulse-program applies stimulated echoes using bipolar gradient pulses for diffusion and 3-9-19 pulses with gradients for water suppression. For each free induction decay, 512 transients were collected with 2 s relaxation delay and a 125 µs delay for binomial water suppression. For all experiments, 8192 data points were collected in the F2 dimension (20 ppm) and 32 data points (gradient strengths) in the F1 dimension. Final data sizes were 8192×256. Exponential multiplication was applied in F2 with 1 Hz line broadening. The diffusion time (Δ) and the gradient length (δ) were set to 200 ms and 2 ms, respectively, while the recovery delay after gradient pulses was 100 µs. For automatic 2D-processing of the raw experimental data, the standard 2D DOSY processing protocol was applied in XWINNMR software with logarithmic scaling in the F1 (diffusion coefficient) dimension. The diffusion coefficient (D) was employed in the Einstein-Stokes relation:
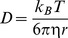
where k_B_ is Boltzmann’s constant, T is the temperature and η is the viscosity of the liquid (2.195 mPa·s for DMSO-*d*_6_ at 25°C [13]) to obtain the hydrodynamic radius of the molecule r (assuming a spherical shape). Subsequently, the following equation was used to calculate the molecular weight:



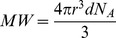
where d is the density and N_A_ Avogadro’s constant.

High resolution – Magic Angle Spinning (HR-MAS) NMR measurements on NM isolated from PUT were recorded on a Bruker AVANCE 400 spectrometer equipped with a 4 mm inverse ^1^H-^13^C HRMAS probe operating at 400.13 MHz and with a Bruker Cooling Unit for temperature control. Samples were spun at 5 kHz, and three different types of 1D proton spectra were acquired using sequences implemented in the Bruker software: (i) a standard sequence with water presaturation during relaxation delay, 10 kHz spectral width, 6400 scans, (ii) a water-suppressed spin-echo T2-filtered Carr–Purcell–Meiboom–Gill [14] (CPMG) pulse sequence to emphasize the mobile components of the sample with water presaturation during relaxation delay with D = 2 s to allow T1 relaxation, 0.3 ms echo time (τ) to permit the broad signals attenuation (‘T2 filter’) and refocusing of spin-coupled multiplets, n  =  a fixed loop of 256 cycles, giving a total spin–spin relaxation delay (2nτ) of 153.6 ms, 8 kHz spectral width, 2560 scans, and (iii) a sequence for diffusion measurements to accentuate components with low diffusion rates based on stimulated echo and bipolar-gradient pulses with Δ 80 ms, eddy current delay 5 ms, δ 2*1 ms and 30% sine-shaped gradient, followed by a 200 µs delay for gradient recovery, 8 kHz spectral width, and 1024 scans.

Solid state MAS ^13^C NMR of NM isolated from PUT was recorded on a Bruker AVANCE 700 NMR spectrometer equipped with a double resonance solid-state 4-mm MAS probe. The sample was inserted into a 4-mm zirconia rotor with teflon insert (sample capacity 12 µL) and spun at 14 kHz, maintaining a constant temperature of 275 K. The following typical settings for direct polarization spectra were used: spectral width 300 ppm, relaxation delay 15 ms, with 1280 and 8192 scans for zg and ^1^H high power decoupled spectra, respectively. ^13^C chemical shifts were referenced to the carboxyl signal at 176.04 ppm of standard glycine.

## Results

### Amino Acid Content of the Pigments

The results of the amino acid analysis of the NMs from the various brain areas (isolated after treatment with proteinase K) are reported in Table 1. The proteinase K treatment is necessary to remove proteins that associate to the NM during the purification process. Liquid chromatography-tandem mass spectrometry peptide analysis of tryptic digests of NM isolated with and without proteinase K proved that this treatment does not remove covalently bound proteins (data not shown). The amino acid content was found to be similar for the NMs of all brain regions, with an average amount of 119.8±8.9 µg/mg of isolated pigment (mean ± sem; n = 22).

**Table 1:** Amino acid content (µg/mg of isolated pigment) ± standard error of the mean (sem).

	CAB-NM(3) a	CAL-NM(2)	CAU-NM(2)	CAX-NM(3)	PAL-NM(2)	PUT-NM(4)	SAB-NM(2)	SN-NM(3)
Aspartic Acid	8.7±2.2	5.6±2.0	6.1±0.5	8.8±0.6	9.1±1.7	12.1±0.3	12.5±1.8	7.1±2.0
Threonine	6.2±1.9	3.1±1.0	4.1±0.4	5.8±0.4	5.1±0.9	7.3±0.5	6.8±1.0	3.5±1.0
Serine	7.1±1.9	4.8±1.7	5.2±0.4	7.4±0.6	7.2±1.0	10.3±0.5	11.1±1.9	5.0±1.4
Glutamic Acid	11.0±2.3	12.4±4.7	6.6±0.4	14.0±1.1	16.9±3.0	17.0±0.2	28.3±5.0	12.1±3.4
Proline	3.7±0.6	5.1±0.2	2.5±0.3	4.9±0.8	5.9±1.0	5.4±0.2	8.0±0.7	3.7±1.1
Glycine	4.0±0.9	2.5±0.7	2.5±0.1	4.6±0.2	4.5±0.8	5.4±0.1	4.9±0.8	4.5±1.2
Alanine	3.3±0.9	2.4±0.6	2.5±0.2	3.9±0.2	3.9±0.7	4.9±0.3	4.4±0.7	3.0±0.8
Cystine	13.8±3.3	4.2±0.7	11.5±1.4	10.3±1.3	8.4±1.6	17.8±2.1	9.0±0.1	6.8±1.7
Valine	6.4±2.3	2.9±0.7	4.3±0.5	6.5±0.4	5.1±0.8	7.7±0.9	5.7±1.5	3.8±0.8
Methionine	1.9±0.5	1.1±0.0	1.1±0.2	2.1±0.1	1.5±0.0	2.0±0.2	1.6±0.6	1.6±0.3
Isoleucine	4.5±1.2	5.4±2.2	2.5±0.2	5.8±0.2	7.3±1.4	6.4±0.4	11.4±1.9	4.0±0.9
Leucine	9.7±3.0	5.0±1.4	6.2±0.7	10.2±0.3	8.7±1.7	12.3±0.9	10.3±1.9	6.7±1.7
Tyrosine	2.9±0.5	2.8±0.9	0.7±0.3	4.1±0.5	2.6±0.3	3.7±0.2	7.2±1.5	3.0±0.8
Phenylalanine	9.7±2.5	6.9±2.7	5.4±0.7	10.9±0.8	10.8±2.2	11.7±0.7	15.9±2.6	6.6±1.8
Lysine	7.8±1.6	12.3±5.2	4.8±0.2	11.5±1.0	14.9±2.5	13.1±0.6	28.9±5.2	8.3±2.2
Histidine	2.3±0.7	3.3±1.1	1.4±0.0	3.0±0.1	3.5±0.6	3.7±0.4	6.5±1.4	2.9±0.6
Arginine	7.9±2.5	5.4±2.0	5.2±0.4	11.9±3.1	7.4±1.2	10.4±0.9	12.8±2.1	5.5±1.3
Total b	110.8±28.6	85.0±27.9	72.4±6.9	125.7±5.9	123.1±21.3	151.1±8.7	185.2±30.6	88.0±23.0

a In parentheses the number of analyzed samples per brain area.

b Mean of total amino acidic content of each sample (each value in this table has been approximated to the first decimal place).

### Elemental Analysis

Elemental analysis of the NM pigments from the different brain areas showed striking similarities between the NMs from CAB, CAU, CAX, SAB, PAL and PUT, whereas the NMs from SN and CAL have significantly different compositions (see Table S1). Elemental analysis was not performed on LC-NM due to insufficient amounts of biological sample. To simplify the calculations on the elemental analysis data, a distinction was made between SN-NM, CAL-NM and other-NMs (encompassing the NMs from the remaining brain areas). Elemental compositions of the three different types of NM are shown in Table 2. The difference in C/N ratio between SN-NM and other-NMs indicates that a higher amount of lipids and a lower amount of melanin is present in the NM from the other brain regions. CAL-NM is characterized by very low nitrogen and sulphur contents compared to the other areas, and appears to contain a very high amount of lipids. Also, the high oxygen content of this area suggests the presence of either high amounts of oxidized material, or large quantities of (incorporated) H_2_O. Although CAL does not contain neurons, instead it contains mainly fibers and glial cells, which can also accumulate NM during aging. The presence of many myelinated fibers in this brain region can increase the amount of myelin lipids contained in the NM isolated from CAL. When this brain region undergoes exactly the same extraction procedure as the other regions, a NM-like substance is obtained. The substance is lighter in color and seems to have slightly different characteristics, nevertheless CAL-NM has been subjected to the same analyses as the other NMs, in order to better define their similarities and differences. Based on the elemental composition, the following average molecular formulas (normalized for S) were deduced (mMW minimum molecular weight):

**Table 2:** Elemental composition and C/N ratio of NM of SN, CAL and other brain areas a.

%	SN-NM	Other-NM	CAL-NM
C	52.81	60.03	52.17
H	6.47	7.92	7.12
N	7.33	5.59	3.42
S	2.93	2.91	1.43
O	24.62	19.30	31.19
C/N	7.2	10.7	15.3

a Average of 2–9 samples per brain area.

SN-NM: C_48.1_H_70.2_N_5.72_O_16.8_S (mMW = 1029.4), with H/C molar ratio = 1.46.

other-NM: C_55.1_H_86.1_N_4.35_O_13.1_S (mMW = 1051.1), with H/C molar ratio = 1.56.

CAL-NM: C_97.4_H_158.3_N_5.47_O_43.7_S (mMW = 2137.2), with H/C molar ratio = 1.63.

The average protein content for all NMs of 119.8 µg aa/mg, obtained by amino acid analysis, was used to calculate the ratio of the different components. Using the empirical formula of C_3.7_H_5.9_NO_1.2_S_0.031_ (C_120_H_191_N_33.5_O_37.8_S when normalized for S) for the protein part, based on the normal distribution of amino acids in human proteins [15], this resulted in residual elemental compositions of C_42.8_H_61.7_N_4.23_O_15.1_S_0.95_ for SN-NM, C_49.6_H_77.4_N_2.83_O_11.4_S_0.95_ for other-NMs and C_86.3_H_140.6_N_2.37_O_40.2_S_0.91_ for CAL-NM after subtraction of the corresponding protein component. Of interest are the different nitrogen/sulphur molar ratios of the three NM types after subtraction of the protein component: 4.45 for SN-NM, 2.98 for other-NMs and 2.60 for CAL-NM. These ratios suggest that the composition of the melanin component might be different for each NM type, with higher amounts of pheomelanin for other-NMs and CAL-NM compared to SN-NM. Previously, a eumelanin/pheomelanin ratio of 3/1 was established for SN-NM [16], but recently new data have emerged suggesting the actual pheomelanin content is higher (unpublished data). For the other NMs this ratio has yet to be determined. If we consider the eu/pheo ratio to be flexible, using the standard formulas of C_8_H_4_NO_2_ (dihydroxyindole) and C_11_H_8_N_2_O_3_S (benzothiazole) as building block units for eumelanin and pheomelanin, respectively, the elemental analysis results suggest the following eu/pheo ratios: 2.45/1 for SN-NM, 0.98/1 for other-NM and 0.6/1 for CAL-NM. If we assume that all remaining sulphur and nitrogen content derives from the melanic part and subtract the appropriate theoretical molecular formula, this would result in the following melanin percentages: 56% for SN-NM, 35% for other-NM and 14% for CAL-NM. Since the lipid component consists mainly of dolichol [17] (C_100_H_164_O), further subtractions based on carbon content lead to lipid percentages of 18% (SN-NM), 42% (other-NM) and 47% (CAL-NM). The calculated percentages of the various components are summarized in Table 3.

**Table 3:** Theoretical percentage a of the various components in SN-NM, other-NM, and CAL-NM.

%	SN-NM	Other-NM	CAL-NM
Melanin	55.9	35.3	14.2
Lipid	18.4	41.7	46.6
Protein	12.0	12.0	12.0
Remaining	13.7	11.0	27.2

a As calculated from elemental analysis data considering varying eu/pheo ratio for each NM type and assuming all nitrogen and sulphur derives from melanin.

However, it is possible that not all sulphur and nitrogen derive from pheomelanin benzothiazines, i.e. the building blocks that are generally considered for the structure of the polymer. Previously obtained results from ultra violet spectroscopy, electron paramagnetic resonance (EPR) spectroscopy and chemical degradation studies indicated a much lower melanin content for SN-NM (10–12%) [2], [16], [18], [19], which leaves substantial amounts of unexplained nitrogen and sulphur. The lipid component of the pigment has negligible amounts of these elements, therefore it appears that some other organic structure is present in the pigment. This possibility will be addressed in detail in the discussion section.

### Infrared Spectroscopy

**Figure 2:**
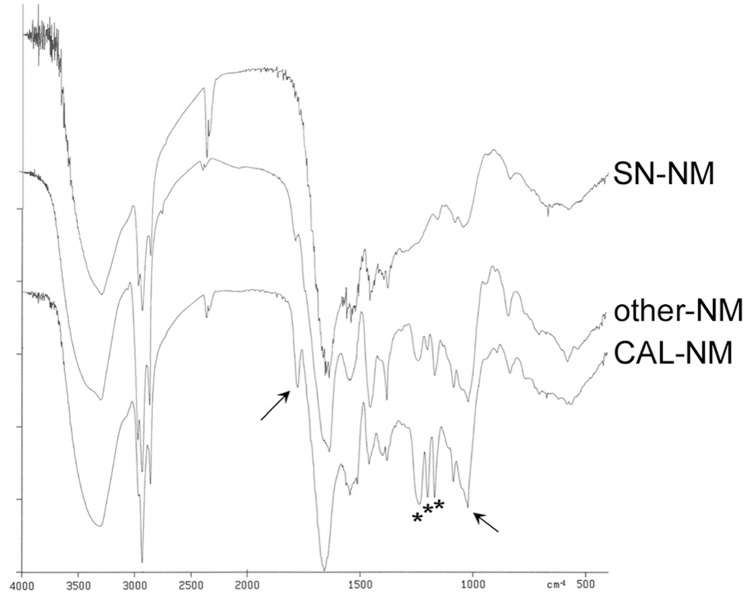
IR spectra of the three NM types. Comparison of IR spectra of SN-NM, other-NM (from PAL) and CAL-NM. Typical absorptions are indicated by asterisks (1230, 1194, 1163 cm^−1^) and arrows (1774, 1015 cm^−1^), for assignments see text.

Despite the significant difference in the elemental analysis results between CAL-NM and other-NMs, their IR spectra are nearly identical (see Figure 2 and Figure S1). Only the relative intensities of certain peaks vary (see below). Surprisingly, both SN-NM and LC-NM show a quite different pattern in the fingerprint region of the spectrum (1500–500 cm^−1^), lacking several characteristic peaks that are present in the spectra of the other-NMs and CAL-NM. Our SN-NM spectrum is similar to previously published IR spectra [20], thus confirming that this NM should be considered separately from the other NMs.

Among the other-NMs and CAL-NM, the IR spectrum of PAL-NM was chosen as representative to be compared to those of SM and SM containing 5% Fe, BSA and SM containing 10% BSA and the lipids extracted from NM with methanol and hexane (Figure S2A–C). A detailed assignment of the IR absorption bands of NM as well as their corresponding components is shown in Table 4. The most striking feature of the IR spectra of the other-NMs and CAL-NM is the presence in the fingerprint region of three intense absorption bands at 1230, 1194 and 1163 cm^−1^ (indicated by asterisks in Figure 2), which are especially intense in the case of CAL-NM. Although the relative intensities of these peaks change for the different brain regions, their positions never vary. Initially, these absorptions proved quite difficult to assign, as they did not seem to derive from any of the known components. Although both the 1230 and 1163 cm^−1^ frequencies seem to be present also in the protein (i.e., BSA) IR spectrum, they are not as intense and pronounced as for NM, and even less so for the SM-protein model in which the protein constitutes ∼10% of the polymer, comparable to the biological sample. Further consideration led to the possible assignment of the 1230 cm^−1^ and 1079 cm^−1^ peaks to PO_2_ asymmetric and symmetric stretching, respectively, from phospholipids, while the peak at 1163 cm^−1^ could (at least in part) derive from either (C = O)-O-C or (C = O)-O-P asymmetric stretching [21], [22], [23]. This explains why these absorptions are also present in the IR spectrum of BSA, which strongly binds (phospho)lipids, that can only be removed by extensive defatting procedures [24], [25]. The presence of phosphorus in the purified NM pigment was confirmed by elemental analysis, which showed a phosphorus content of 0.17%. Although it was not possible to perform ^31^P-NMR on the NM pigment due to its low solubility, the ^31^P-NMR spectrum of the lipid extracts of NM confirmed the presence of several phospholipids (data not shown).

**Table 4:** Detailed assignment of the IR absorption bands of NM.

cm^−1^	assignment	component
3031	= C-H stretching	Lipid
2950	CH_3_ asymmetric stretching	Lipid
2925	CH_2_ stretching	Lipid
2853	CH_3_ symmetric stretching	Lipid
1450	CH_2_-CH_3_ bending	Lipid
1376	CH_3_ symmetric vibration	Lipid
832	= CH bending tri-substituted	Lipid
1515	C = N stretching	Melanin
1400	C-N amine stretching	Melanin
1285	possibly phenol C-OH stretching	Melanin
1542	N-H bending, C-N stretching amide II	Protein
932	O-H bending and/or C-O-C vibration	Protein
3400	O-H stretching	Melanin + Protein
3286	N-H stretching	Melanin + Protein
1630	C = O stretching	Melanin + Protein
1774	C = O stretching	(ganglioside?) lactone
1230	PO_2_ asymmetric stretching	Phospholipids
1194	C-O stretching	(ganglioside?) lactone
1163	CO-O-C or CO-O-P asymmetric stretching	Phospholipids
1079	PO_2_ symmetric stretching	Phospholipids
1015	C-O stretching	(ganglioside?) lactone

Other additional IR peaks that could not be explained by any of the separate components are those occurring at 1774 and 1015 cm^−1^ (indicated by arrows). Based on their positions, these absorption peaks were tentatively assigned to C = O and C-O stretching, respectively, (also at 1194 cm^−1^) from lactone structures, possibly from ganglioside lactones which are present in sphingomyelin and are known to accumulate with aging [26]. The absence of these NM-specific IR peaks in the spectra of lipid extracts of NM in organic solvents might be explained by the fact that the hydrophilic sugar and phosphate moieties prevent the solubilization in organic solvents.

The IR spectra of both SN-NM and LC-NM are quite different from those of the other NMs because they lack the typical absorption bands at 1774, 1230, 1194 and 1015 cm^−1^, while the 1163 and 1079 cm^−1^ peaks are much less pronounced. If the previous assignments of these peaks are correct, SN-NM and LC-NM seem to contain much less phospholipids and (ganglioside) lactones than the other NMs. This partly coincides with the elemental analysis results which suggest that SN-NM contains only about 18% lipids, *vs.* 40% for other-NMs.

### Nuclear Magnetic Resonance - Solution

**Figure 3:**
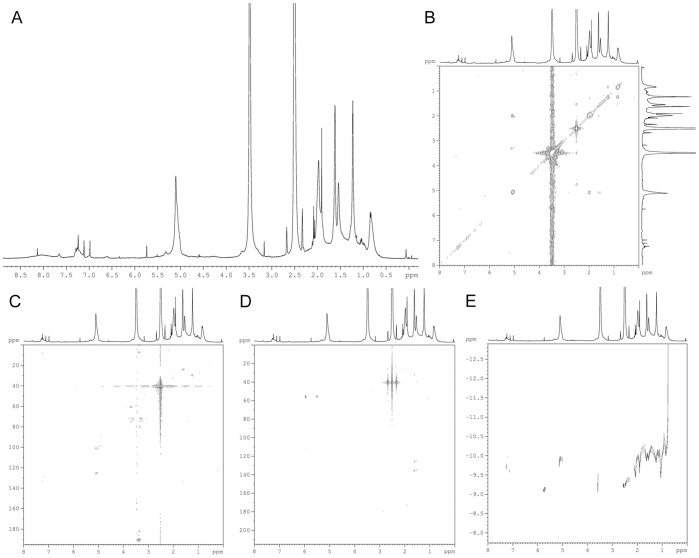
1D- and 2D-NMR spectra of NM. Representative NMR spectra of NM, those shown are for the NM isolated from CAB: (A) 1D proton, (B) COSY, (C) ^1^H-^13^C HSQC, (D) ^1^H-^13^C HMBC, (E) DOSY.

NM is sparingly soluble in DMSO. High resolution solution NMR spectra (1D proton, 2D-COSY, 2D ^1^H-^13^C HSQC and HMBC) of all NMs confirmed the presence of a dolicholic structure [17]. Representative spectra of each NMR experiment are shown in Figure 3, additional 1D proton spectra for all NMs are shown in Figure S3. Typical ^1^H dolichol-related signals occur at 1.6 (-CH_3_), 2.0 (-CH_2_-), and 5.1 ( = CH) ppm, with corresponding ^13^C signals that are shifted slightly (mostly downfield, except for the = CH carbon signal) in comparison to standard dolichol values [27] (see Table 5).

**Table 5:** Experimental ^13^C ppm values of NM pigments as determined by 2D ^1^H-^13^C HSQC, compared to known values of bovine liver dolichol [27].

Group	^1^H (ppm)	^13^C_NM_	^13^C_dolichol_
-CH_3_	1.6	23	18
-CH_2_	2.0	27, 32	21, 26
= CH	5.1	101, 125	127
>C =	*^a^	135	131

a Quaternary carbon atom as determined by HMBC NMR.

Since the dolichol-related signals persist even after extensive washing of the pigment with different types of organic solvents, it can only be concluded that the dolichols are covalently bound to the NM structure. The type of binding is not ester-like, since heating of NMs in potassium hydroxide does not result in the formation of free dolichols (data not shown). Compared to dolichol, the NM pigment has an additional ^13^C signal at 101 ppm which is correlated with the dolichol 5.1 ppm ^1^H peak. This indicates that the binding of the lipid to the melanin polymer is likely to occur at the –CH_2_- position of dolichol, causing an upfield shift of the neighbor carbon atom signal. Another indication for this hypothetical binding site is the intensity ratio between the signals at 2.0 and 1.6 ppm, which is ∼1.1 for the other-NMs, compared to 1.3 for dolichol. The exceptions are, again, the NMs from SN and CAL. In both cases the 2.0/1.6 ppm peak intensity ratio is lower than for the other NMs (i.e. 0.2 and 0.8 *vs.* 1.1, respectively), as is also the 2.0/5.1 ppm intensity ratio (i.e. 0.5 and 0.2 *vs.* 2.1, respectively). However, the intensity ratio between the peaks at 1.6 and 5.1 ppm is similar for SN-NM and other-NMs (2.4 *vs.* 2.1), whereas for CAL-NM it is significantly lower (0.2). Although the elemental analysis suggests that CAL-NM contains a much higher lipid content than the other NMs, the low 2.0/5.1 ppm and 1.6/5.1 ppm intensity ratios indicate a high level of unsaturation; therefore, simple dolichol is likely not the main lipid component. This result is in accordance with previously published results of lipid analysis from the NMs of the various brain areas [28], which showed that the lipid extracts of SN-NM and other-NMs contained ∼25% and ∼10% dolichol/dolichoic acid, respectively, whereas the lipid extract of CAL-NM contained only ∼1% dolichol. While the nature of the main components of CAL-NM lipids has not yet been clarified, it is clear from both the ^1^H NMR spectrum and the elemental analysis results that these lipids are highly unsaturated, and possibly oxidized. For SN-NM the situation is different, as the 1.6/5.1 ppm intensity ratio is very similar to that of other-NMs. Since dolichol is the main lipid component, the differences in the 2.0/1.6 ppm and 2.0/5.1 ppm ratios can not be explained by the presence of other lipids. Instead, the weak dolichol signal at 2.0 ppm is likely due to highly substituted C-H (because of binding to the melanic and/or proteic components) and possibly oxidized, as indicated by the higher percentage of oxygen in the elemental analysis data.

Long range ^1^H-^13^C heterocorrelated NMR spectra provided additional insight into the pigments’ composition. The dolichol quaternary carbon atom is found at 135 ppm (long range interaction with the ^1^H 1.6-ppm signal), slightly shifted downfield compared to free dolichol. All interactions derived from the two-dimensional spectra are summarized in Table 6. The aliphatic signals at 1.2 and 0.8 ppm show no COSY interactions with the dolichol signals, therefore they must derive from other lipids. The corresponding ^13^C signals at 30 and 28 ppm, respectively, confirm that these are saturated aliphatic carbon atoms. The sharp 1.9-ppm ^1^H signal, which is present in almost every NM with variable intensity, shows 21-ppm HSQC and 173-ppm HMBC cross peaks therefore likely derives from acetate groups, possibly from acetylated amino acid residues [29].

**Table 6:** Summary of COSY, HSCQ and HMBC interactions a.

^1^H (ppm)	COSY (ppm)	HSCQ (ppm)	HMBC (ppm)
7.2	-	128*; 121*	149*; 43*
5.1	2.0; 1.6*	125; 101*	-
2.0	5.1; 1.6*	32; 27*	-
1.9	-	21*	173*
1.6	5.1*; 2.0*	23*	135*; 125*; 32*
1.2	0.85	30	-
0.85	1.2	28*	-

a The asterisks (*) indicate the interaction was not observable in all NMs spectra, possibly due to low concentration of the pigment.

The ^1^H-NMR spectra of several (but not all) NMs showed additional peaks at 3.7, 4.6 and 5.5 ppm, besides the lipid signal at 5.1 ppm. Both the signals at 4.6 and 5.5 ppm showed ^1^H/^1^H interaction with the 3.7 ppm signal, but had no corresponding ^13^C signals in the heterocorrelation spectrum, which indicates that these protons are not carbon-bound, and therefore are likely bound to N or O atoms. The 3.7 ppm signal had corresponding ^13^C signals at 61 and 74 ppm. Repeated extractions of NM with DMSO eliminate these signals from the spectrum while the dolichol related signals remain (data not shown). Therefore, they do not seem to be due to groups covalently bound to the pigment, and probably derive from proteic material that remains associated to the pigment during the isolation procedure.

Unfortunately, the melanic part of NM is not detectable in the solution NMR spectrum. Apart from being only slightly soluble, the extended polymeric structure likely contains very few protons, and therefore does not produce signals of detectable intensity. The signal at 7.2 ppm disappears after 2–3 extractions with DMSO, indicating that it is not due to a group covalently bound to the pigment and therefore is not to be considered as deriving from the melanic part of NM.

### Diffusion

Diffusion measurements are quite useful to determine which signals in the 1D NMR spectrum actually belong to NM, and which should be considered to result from contaminating by-products. In fact, the poor solubility of the pigment in DMSO requires very long acquisition times, which magnify even trace substances present in the sample or the solvent that might be erroneously attributed to NM (i.e. the signals at 6.5, 8.2 and 8.5 ppm). Also, the signals at 3.7, 4.6 and 5.5 ppm, that were previously attributed to lipid material other than dolichol, can now be assigned to non-covalently bound (proteic) material with higher diffusion constants, and therefore lower molecular weight, than NM.

Molecular weight determination with DOSY-NMR is sensitive to the shape and relaxation properties of the molecules, as well as other solution conditions. Therefore, the accuracy of molecular weight determination by DOSY-NMR cannot be compared to high-resolution methods like mass spectrometry. However, since mass spectrometry is not an option for the heterogenous and largely insoluble NM polymer, and as far as we know no molecular weight for NM has yet been established, DOSY-NMR seems the best technique available to obtain an indication of the molecular size of the pigment. Solution diffusion measurements of the various NMs provided a range of diffusion coefficients between 1.20×10^−10^ m^2^/s and 3.55×10^−11^ m^2^/s for the lipid 5.1 ppm signal, corresponding to diameters of 16 and 55 Å and molecular weights of 1.4 and 52 kDa respectively (as calculated for spherical molecules). Since the most common form of dolichol (19–21 isoprenic units) has a molecular weight of ca. 1.3 kDa, this result indicates that the DMSO-soluble part of NM likely consists of a mixture of dolichol molecules substituted with small melanic and/or peptide oligomers, and larger polymers consisting of several lipid molecules bound to other components. The part of the pigment that is insoluble in DMSO is expected to consist of heavily substituted extended polymers of higher molecular weight.

### HR-MAS NMR

HR-MAS NMR combines the typical advantages of solid-state and solution NMR techniques. This technique employs the magic angle spinning to minimize the dipolar coupling and chemical shift anisotropy effects, which give rise to the broadening of signals due to restricted molecular motion. The use of CPMG pulse sequence in HR-MAS NMR analysis minimizes the broadening of resonance signals due to high molecular weight components, thus emphasizing the more mobile components [14]. The diffusion-edited spectrum allows the observation of components with low diffusion rates and large line width.

**Figure 4:**
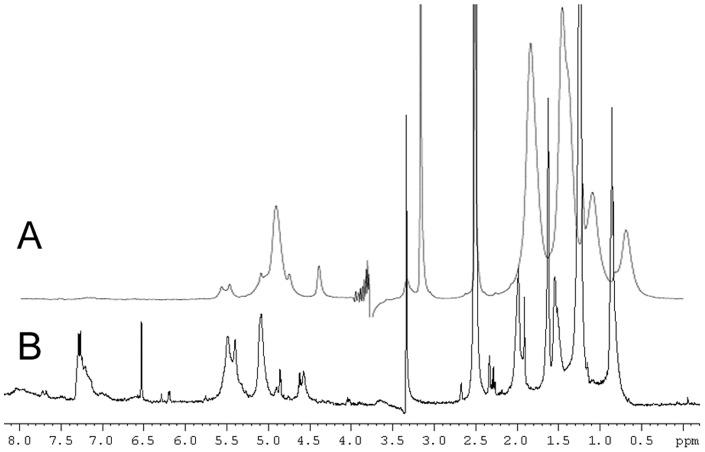
1D proton HR-MAS and solution NMR spectra of NM from PUT. (A) HR-MAS water-presaturation pulse sequence with composite pulse; (B) solution spectrum with water suppression by gradient-tailored excitation. In the HR-MAS spectrum most major signals are shifted slightly upfield.

**Figure 5:**
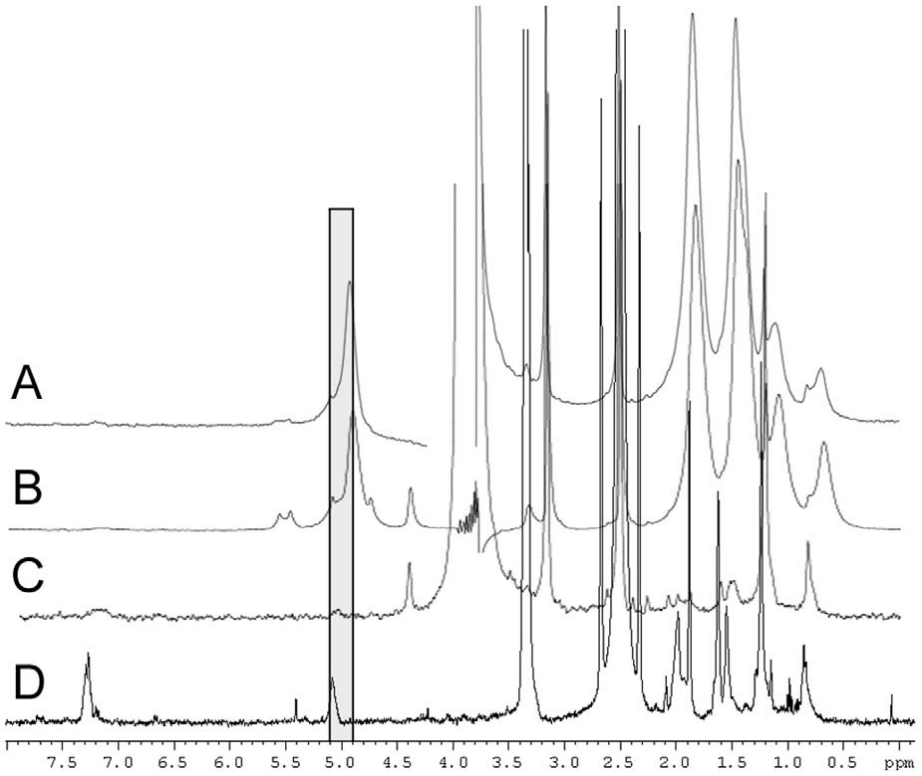
1D proton HR-MAS NMR spectra of NM from PUT. (A) HR-MAS diffusion-edited spectrum; (B) unfiltered HR-MAS spectrum; (C) HR-MAS CPMG sequence; (D) solution CPMG sequence. The vertical grey box shows the presence of two different signals around 5.0 ppm.

In the NMR spectrum of NM from PUT acquired using HR-MAS (Figure 4) most major signals were slightly shifted (mostly upfield) compared to the solution spectrum. Whereas in the solution spectrum the aliphatic signals at 0.8 and 1.2 ppm are more intense than the dolichol-related signals at 1.6 and 2.0 ppm, in the HR-MAS spectrum the situation is reversed, indicating a slightly different composition of the insoluble part of the pigment compared to the DMSO-soluble part. The HR-MAS spectrum shows that the dolicholic signal around 5.0 ppm consists of two different components, a main peak at 4.9 ppm flanked by smaller peaks at 5.1 and 4.7 ppm, the latter of which deriving from the 4.8 ppm peak of the solution spectrum. The 5.1 ppm peak corresponds to the principal component of the ^1^H solution spectrum, as was shown by the CPMG (T2-filtered) spectra (Figure 5C, D), in which the larger component completely disappears, while the soluble component remains visible. Apparently, the DMSO-soluble part of NM represents only a small percentage (∼5%) of the pigment. The diffusion-edited spectrum (Figure 5A) shows a main component at 4.9 ppm, identical to the main component of the unfiltered spectrum (Figure 5B). This signal was absent in the solution spectrum, and it can therefore be concluded that it is due to a less mobile and completely insoluble lipid component. The mobility likely depends on the number of substitutions on the lipid chain, where the presence of more binding sites to the melanin polymer results in reduced mobility.

**Figure 6:**
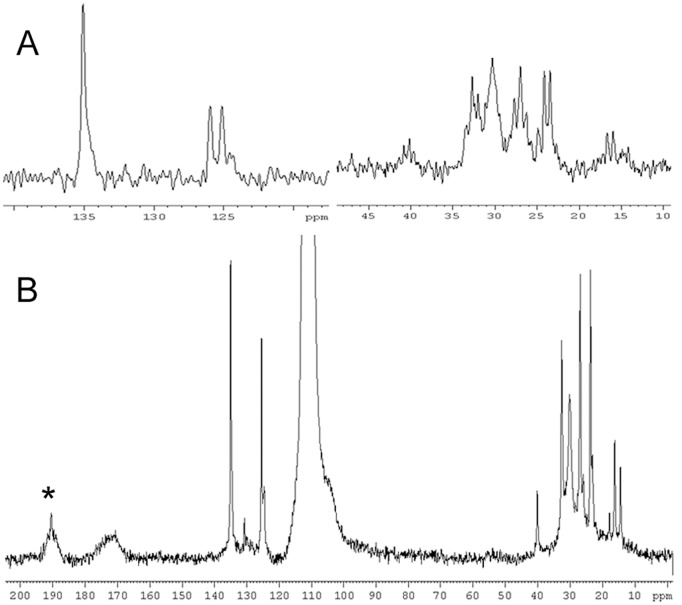
Solid state ^13^C NMR spectra of NM from PUT. (A) regions of interest in the *zg* spectrum; (B) high power decoupled spectrum. The intense signal at 110 ppm derives from the teflon insert, * indicates the spinning side band. The second (right hand) spinning side band (at ∼ 30 ppm) disappears beneath sample signals.

It was not possible to obtain reliable COSY or HSQC/HMBC spectra for the HR-MAS sample due to the small amount of sample and the low sensitivity of these sequences. However, the TOCSY experiment, showing correlations between all protons in a spin system (not just those directly coupled to each other as in the COSY experiment), is much more sensitive. In the TOCSY sequence the fundamental parameter is the mixing time, during which magnetization is transferred to coupled spins. Since the T2 is very short, especially for the insoluble part of the NM pigment, it was found that 2 different mixing times (20 ms and 60 ms) were needed to develop the cross peaks previously observed in the solution COSY spectra. In fact, with a mixing time of 20 ms, the interaction of 4.9–1.8 signals was visible; after a longer mixing time of 60 ms this interaction was lost but new interactions between 1.1–0.7 and 1.45–1.1 signals were observed (spectra shown in Figure S4). By showing the same interactions as found in the solution COSY spectra, the TOCSY experiment demonstrates that, although the relative intensities of some signals may vary, the insoluble part of the pigment has essentially the same structure as the soluble part. The additional interactions 4.4–3.7 and 4.4–3.1 are likely due to a hydrogen bond between NH and H_2_O (also previously observed in COSY), and a multiple bond interaction between NH and the methyl group of MeOH, respectively [29].

### Solid State ^13^C NMR

The primary objective of the ^13^C CPMAS solid state experiment was to obtain quantitative information on the aromatic (melanic) and aliphatic (lipid) carbon ratio, since other analytical and spectroscopic techniques provided conflicting data regarding the amount of melanin in the pigment (see the discussion below). Solution NMR could not clarify this discrepancy, as the melanic part contains very few protons and is therefore almost invisible in the proton NMR spectra.

A quantitative solid state ^13^C NMR spectrum would have been able to solve this issue. Due to the characteristics of our sample, in particular its high mobility, it was not possible to acquire a CPMAS spectrum under our experimental conditions. Although direct polarization techniques can not be considered quantitative, they still provide interesting information regarding the structure of the molecule under consideration. The only drawback in this specific case was the presence of the teflon insert in the reduced volume rotor. Since teflon does not contain protons it does not interfere with CPMAS analysis, but unfortunately it has a rather strong signal at 110 ppm in direct polarization spectra, thus effectively covering a potentially interesting part of the spectrum. However, both the *zg* and the high power decoupled spectra provided additional information about the lipid component of the NM pigment (see Figure 6 and Table 7). Although the melanic component of the pigment is not visible, the lipid signals are much better resolved compared to previously published ^13^C solid state spectra [30], [31]. Because of the high quantity of NM required for the solid state spectrum (∼ 12 mg) and the extended acquisition time, the ^13^C experiment was performed only for NM from PUT, and not for the NMs from the other brain regions.

**Table 7:** Results from the ^13^C solid state NMR spectrum.

^13^C ppm	Protonation state a	^1^H ppm b
173	C = O	1.9 (LR c)
135	C	1.6 (LR)
131	–	–
126	CH	5.1
125	–	–
40	CH_x_ d	–
32	CH_2_	2.0
30	CH_x_	1.2
27	CH_2_	2.0
23	CH_3_	1.6
16	CH_x_	–
14	CH_x_	–

a Carbon protonation state as deduced from the zg spectrum.

b Corresponding ^1^H chemical shift as deduced from heterocorrelation solution spectra.

c LR = long range interaction.

d CH_x_ = proton-bound C, impossible to determine the level of protonation.

## Discussion

### Three Types of NMs can be Distinguished

NM is a highly stable substance contained in lysosomal type organelles which accumulate in neurons of human and animal brain during the entire lifespan. Clarifying structural aspects of NM would explain its high stability, since NM is present in lysosomal organelles with several enzymes like proteases, lipases and oxidative enzymes but it is not degraded. A partial explanation for its stability toward oxidation was given in our previous work, reporting an oxidation potential of the surface of NM granules that is not sufficiently high to induce oxidative reactions [32]. This low oxidation potential of NM is consistent with the model assuming that pheomelanin constitutes an internal core encapsulated by eumelanin in the melanic portion of NM.

Comparison of the elemental analyses of the various NMs enables to identify three distinctive types of NM: SN-NM, CAL-NM and other-NMs (the latter including all NMs from the remaining brain regions). As it was found previously in chemical degradation studies [2], the C/N molar ratios of the three NM types suggest that SN-NM contains a higher amount of melanin compared to other-NMs and is presumably more oxidized, whereas NM from CAL contains particularly large quantities of lipid and has a much smaller melanin component. Also, after subtraction of the protein component, the N/S molar ratios indicate different eumelanin/pheomelanin ratios for each type of NM, varying from highest for SN-NM to lowest for CAL-NM. Assuming that the remaining nitrogen and sulphur content derives exclusively from melanin, a theoretical composition for each NM type was calculated. However, these calculations resulted in a melanin content that is much higher (more than 50% for SN-NM) than was previously obtained in ultra violet spectroscopy, EPR spectroscopy and chemical degradation studies of NM pigment, in which the melanin content was estimated to be 10–12% based on comparison to SM [2], [16], [18], [19]. This discrepancy indicates that, apart from ‘normal’ melanin, another oligomeric structure must be present, containing both nitrogen and sulphur, and probably with an aromatic/heterocyclic nature, since NMR shows no evidence of protons other than those originating from lipids. In other words, something similar to melanin but lacking some of the typical characteristics associated with melanic structures, like the organic radical EPR signal, the characteristic ultra violet absorption spectroscopy and the formation of several known degradation products upon H_2_O_2_ oxidation. A possible explanation is that the unknown structure is derived from melanin upon some kind of aging or maturation process. Recently, studies have emerged showing that the structure of melanin undergoes changes over time. By mimicking the aging process (by heating at different temperatures), it was shown that the initial benzothiazine structure of cysteinyl-3,4-dihydroxyphenylalanine and cysteinyl-DA melanins is slowly converted into the more stable benzothiazole [33], [34]. If the structure of SM can change in time without external influences, it is possible that the melanic component of NM also undergoes some kind of change during aging and as a result it might display different characteristics than newly synthesized melanin. In fact, NM is contained in lysosomal organelles and is constantly in contact with a variety of enzymes and other biomolecules. Therefore, care must be taken when comparing NM properties to those of SM, as the time factor (typically 50-70 years, since NM is mostly collected from older subjects) may have a profound impact on both its structure and properties. In this respect, it should be taken into account that, besides the typically recognized melanic degradation products (pyrrole-2,3,5-tricarboxylic acid, thiazole-2,3,5-tricarboxylic acid, 4-amino-3-hydroxyphenylalanine, etc.) [2], the pattern of compounds resulting from degradation studies of NM includes products that have not yet been characterized. Once identified, these degradation products might explain the apparent discrepancy between the elemental analysis results and spectroscopic studies in the estimation of the melanin content.

### The Synthesis of NMs Starts from Protein Fibrils Followed by Reaction with Catechols, Dolichols and Subsequent Polymerization

Another important consideration is the difference in X-ray scattering studies performed on both SM and NMs [2]. All analyzed NMs showed a peculiar diffraction pattern of *ca.* 4.7 Å, which is quite different from the pattern associated with aromatic stacking sheets with the typical distance of 3.4 Å found for both SM and *Sepia* melanin [35]. The NM diffraction pattern is actually similar to the cross-β sheet structure of amyloid fibrils [36], suggesting that here it is the protein component which is responsible for the organized structure, possibly a β-structured core tightly bound to the melanin polymer. This raises an interesting question about the initial steps of NM formation. Whereas both autoxidation and tyrosinase oxidation of DA lead to the formation of tightly packed melanic polymers, the melanin component of NM is clearly less structurally organized. Therefore, it is likely that NM formation does not result from an enzymatically controlled reaction, but it rather consists in a different process. We propose that NM formation starts with the accumulation of aggregated proteins in the cytosol, resulting in the formation of amyloid fibrils. In fact, preliminary proteomic analysis of both NM pigment and NM containing organelles confirm the presence of proteins that are capable of forming insoluble fibrils, like α-synuclein (unpublished data). The next step in NM synthesis is the formation of adducts between the protein fibrils and excess DA and/or DA metabolites, followed by polymerization to form the melanic component. The resulting undegradable substance is then taken into autophagic vacuoles and transported to the lysosome where it can interact with the lipids (dolichols) present, thus initializing the transformation of lysosome to NM containing organelle.

Indeed, if the initial steps of NM synthesis involve the formation of adducts between protein residues and DA/DOPA quinones, followed by polymerization and formation of covalent bonds with lipids, the resulting structure can not be organized in stacked sheets, as it is for a purely melanic polymer. As a consequence, the g = 2 EPR signal might not reflect the entire melanin content, as the organic radical will be less delocalized when melanic oligomers are surrounded by protein and/or lipid residues. Cross-linking to proteins and lipids is likely to influence also the outcome of NM chemical degradation process, which recognizes easily only the degradation products from the extended aromatic polymer.

The (spectroscopic) techniques that have been applied to date have not been able to completely characterize the nature of NM. Sulphur-specific studies may be needed to identify the various forms in which sulphur is present in the pigment, which is crucial to elucidate the synthetic pathway of NM and its neuroprotective role. As the formation of NM serves to remove reactive/toxic catechols from cytosol [37], characterization of the sulphur moieties in NM could indicate the type of reactive sulphur-catechol derivatives that endanger neurons.

The IR spectra of the other-NMs and CAL-NM were nearly identical, whereas SN-NM and LC-NM showed a quite different pattern in the fingerprint region. The other-NMs and CAL-NM show characteristic peaks at 1774, 1230, 1194 and 1163 cm^−1^, that were tentatively assigned to various stretching vibrations of phospholipids and possibly (ganglioside) lactones. These peaks are particularly intense in the case of CAL-NM, confirming the high amount of lipid material present in this NM. Given the really small amounts of material that can be obtained from the extraction procedures, small contaminations cannot be excluded, although all pigments undergo the same purification procedure. During the isolation process both phospholipids and lactone groups of gangliosides contained in the sphingomyelin, which is a major component of myelin sheaths, could be adsorbed into NM structure and retained even after extensive washing. This explanation is supported by the observation that CAL-NM shows the most intense peaks of phospholipids and lactone groups. In fact the CAL region from which CAL-NM is prepared has the highest number of myelinated fibers and content of sphingomyelin among the brain regions investigated in this work.

### In NMs there is a Soluble Component Having a Lower MW and Dolichol Content Compared to the Insoluble Component

Isolated NM consists of smaller DMSO-soluble components and much larger, completely insoluble components. Although the soluble part is clearly less polymeric than its insoluble counterpart, it should still be considered as part of the NM pigment, since in aqueous environment (as *in vivo*) it is insoluble like the rest of the polymer. The soluble part likely consists of oligomeric precursors of the polymeric NM. Extensive NMR studies on both the soluble and the insoluble part by solution NMR, HR-MAS and solid state ^13^C measurements confirmed that both components have essentially the same composition, although the insoluble part seems to contain more dolichol and less saturated lipids than its soluble counterpart.

The melanic component is extremely difficult to study by NMR. The lack of protons in the polymeric aromatic structure poses severe limitations, whereas the aromatic ^13^C signals probably require extremely long acquisition times to allow for relaxation. NMR studies, therefore, concern mostly the lipid component of the pigment. Dolichol is confirmed to be the main lipid component of SN-NM and other-NMs and is especially prevalent in the insoluble part of the pigment, whereas CAL-NM contains large quantities of highly unsaturated and/or oxidized lipids. DOSY-NMR allowed to determine a range of average molecular weights from 1.4 to 52 kDa for the DMSO-soluble part of the pigment. This indicates that the soluble part of NM is a mixture of dolichols substituted with oligomeric melanic and/or proteic residues and more complex polymeric structures containing covalently bound dolichols. The DMSO-insoluble part likely consists of highly substituted polymeric structures of very high molecular weights. The presence of large amounts of both oligomeric and polymeric structures shows that NM synthesis and accumulation is a continuous process occurring in organelles inside neurons. The protein-melanin core is likely synthesized in the cytosol where oxidative enzymes acting on DA, and DA metabolites, progressively increase the size of melanic oligomers, which are then taken into autophagic vacuoles and transported into lysosomes. Here the melanic oligomers can interact with dolichols released by the lipid bodies present in the lysosome to form the melanic-dolichol adducts. The latter can polymerize to form the insoluble NM.

NM from LC shows IR and NMR spectra that are very similar to SN-NM, which was expected because of their similar behavior in PD. Elemental analysis could not be performed on LC-NM because of insufficient sample, but it is expected that these data follow the same trend.

### Conclusions

Investigations on NMs isolated from different brain areas have shown that they can have a melanic content over 50%. This melanic component is made of the classical melanin structure with aromatic system having a stable free radical and of an oligomeric structure of aromatic heterocyclic type with nitrogen and sulphur.

Based on the presented results, we suggest that NM synthesis starts in cytosol where proteins aggregate to form fibrils which react with catechols to give adducts which can be oxidized and polymerize generating the melanic component. The resulting undegradable protein-melanin compound can be taken into autophagic vacuoles and transported into lysosomes where it will react with dolichols to form the final NM. In fact, it was found that NMs have a soluble component with MW 1.4–52 kDa consisting of a protein-melanin-dolichol system. The insoluble component of NMs has a higher MW with the protein-melanin-dolichol having a higher dolichol content. The dolichols contribute to insolubilize the NM structure. There is a continuous process of NM accumulation in the lysosomal organelle, starting first with formation of soluble NM oligomers followed by further polymerization of the melanic component and reaction with dolichols.

## Supporting Information

Figure S1 IR spectra of NM of the various brain areas.
(TIF)

Figure S2 **IR spectra of NMs compared to spectra of the separate components.** IR spectra of (A) other-NM (from PAL) and SN-NM compared to SM and SM containing 5% Fe (indicated in the figure with SM-Fe), (B) other-NM and SN-NM compared to BSA and SM containing 10% w/w BSA (indicated in the figure with SM-BSA), (C) other-NM and SN-NM compared to lipids extracted from NM with methanol and hexane (NM-lipids). Relevant wavelengths are shown by vertical lines.
(TIF)

Figure S3 **1D proton NMR spectra of all NMs in DMSO-d^6^.**
(TIF)

Figure S4 **HR-MAS-TOCSY experiment of NM from PUT using different mixing times.** (A) mixing time = 20 ms to show crosspeak between 4.9 and 1.8 ppm, and (B) mixing time = 60 ms to show crosspeaks 1.1–0.7 and 1.45–1.1 ppm.
(TIF)

Table S1 **Elemental analysis of NMs of the various brain areas.** This table shows the striking similarities between PUT-NM, CAB-NM, CAX-NM, SAB-NM, PAL-NM and CAU-NM (data averaged as ‘other-NM’).
(DOC)
