# Supplementary figures and images for: Neuromelanins of Human Brain Have Soluble and Insoluble Components with Dolichols Attached to the Melanic Structure
Source: PLoS One. 2012 Nov 5;7(11):e48490. doi: 10.1371/journal.pone.0048490 (PMC3489676; doi:10.1371/journal.pone.0048490)

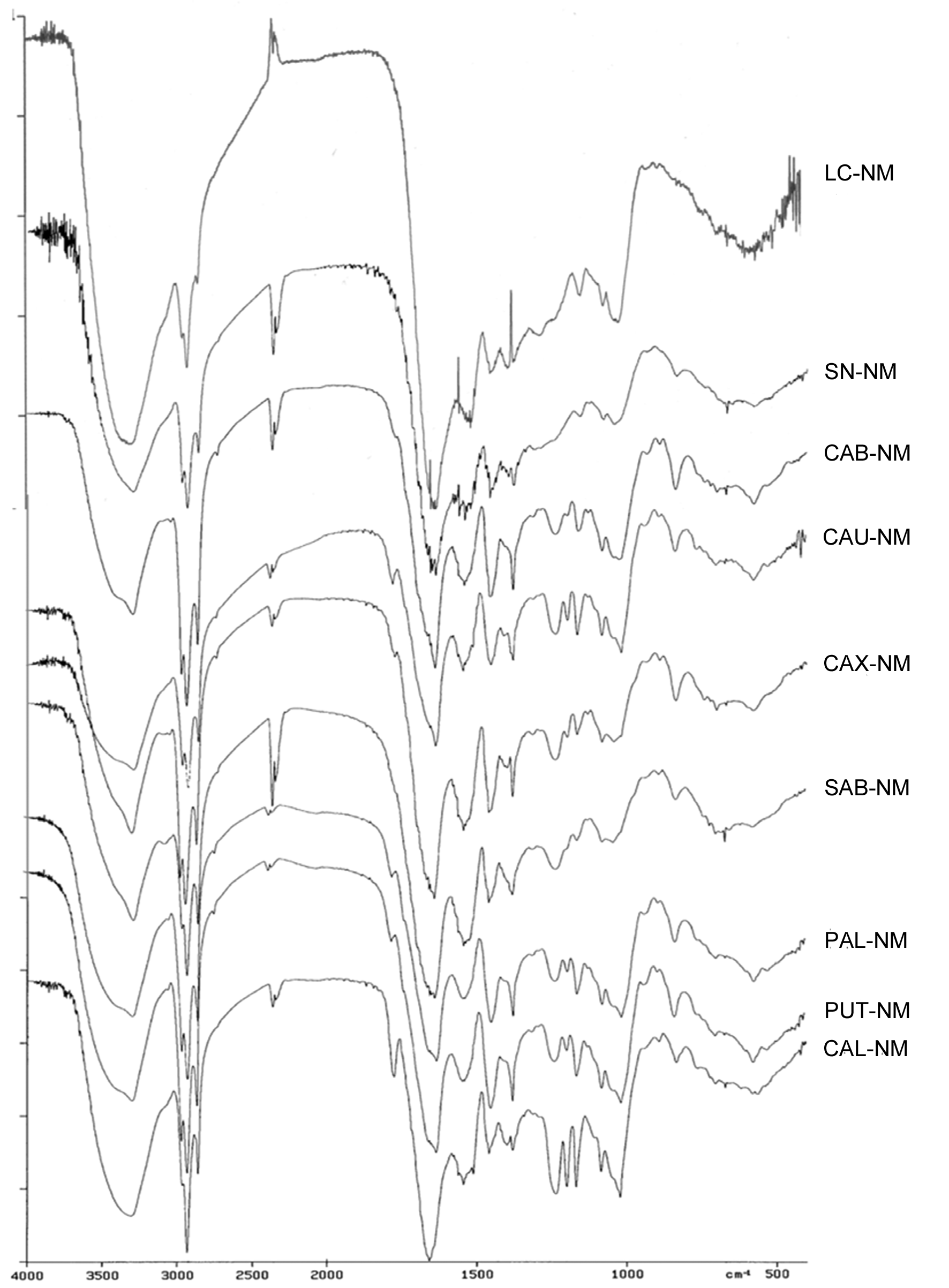

Supplement: Figure S1 — IR spectra of NM of the various brain areas. (TIF) [file pone.0048490.s001.tif]

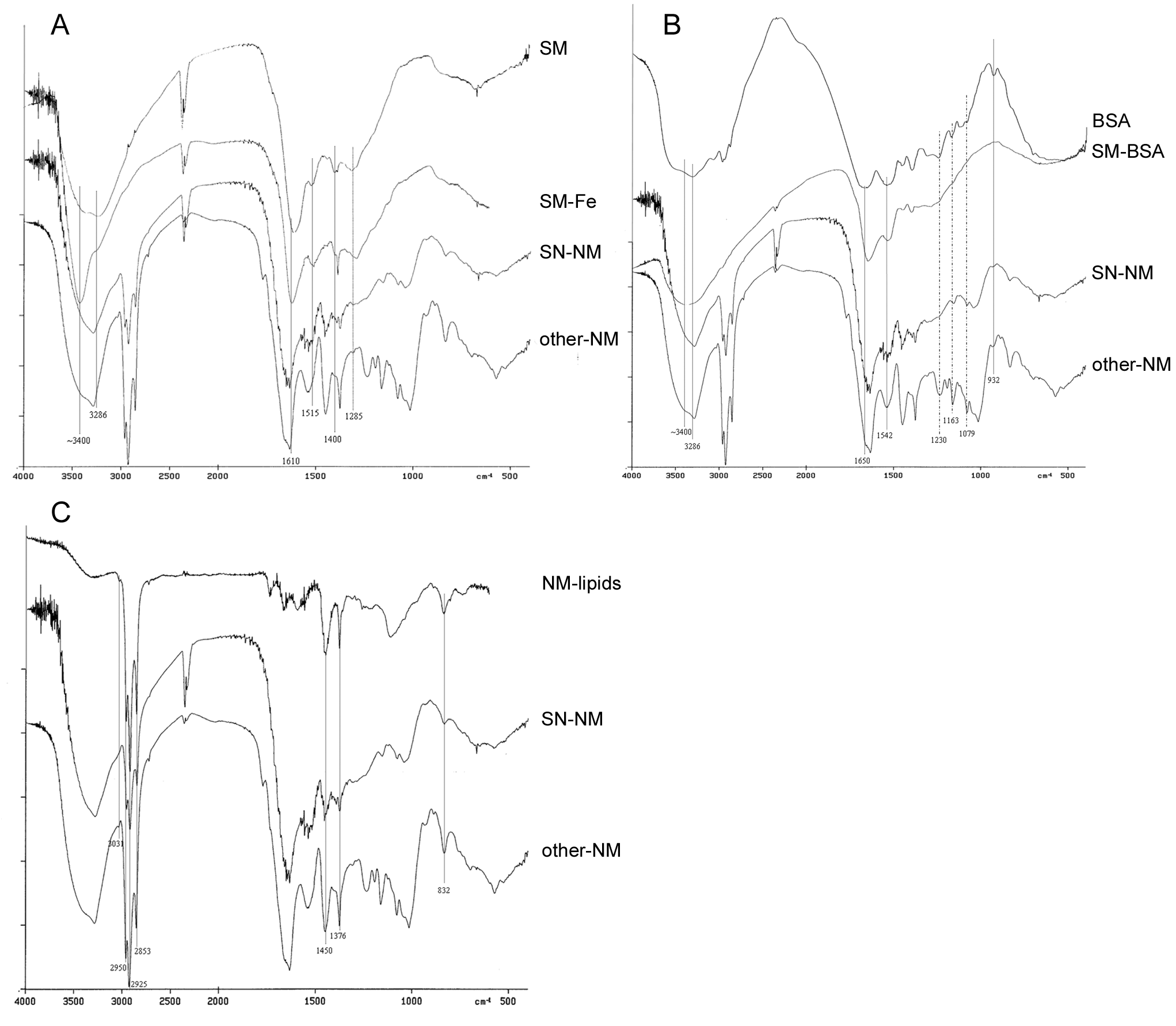

Supplement: Figure S2 — IR spectra of NMs compared to spectra of the separate components. IR spectra of (A) other-NM (from PAL) and SN-NM compared to SM and SM containing 5% Fe (indicated in the figure with SM-Fe), (B) other-NM and SN-NM compared to BSA and SM containing 10% w/w BSA (indicated in the figure with SM-BSA), (C) other-NM and SN-NM compared to lipids extracted from NM with methanol and hexane (NM-lipids). Relevant wavelengths are shown by vertical lines. (TIF) [file pone.0048490.s002.tif]

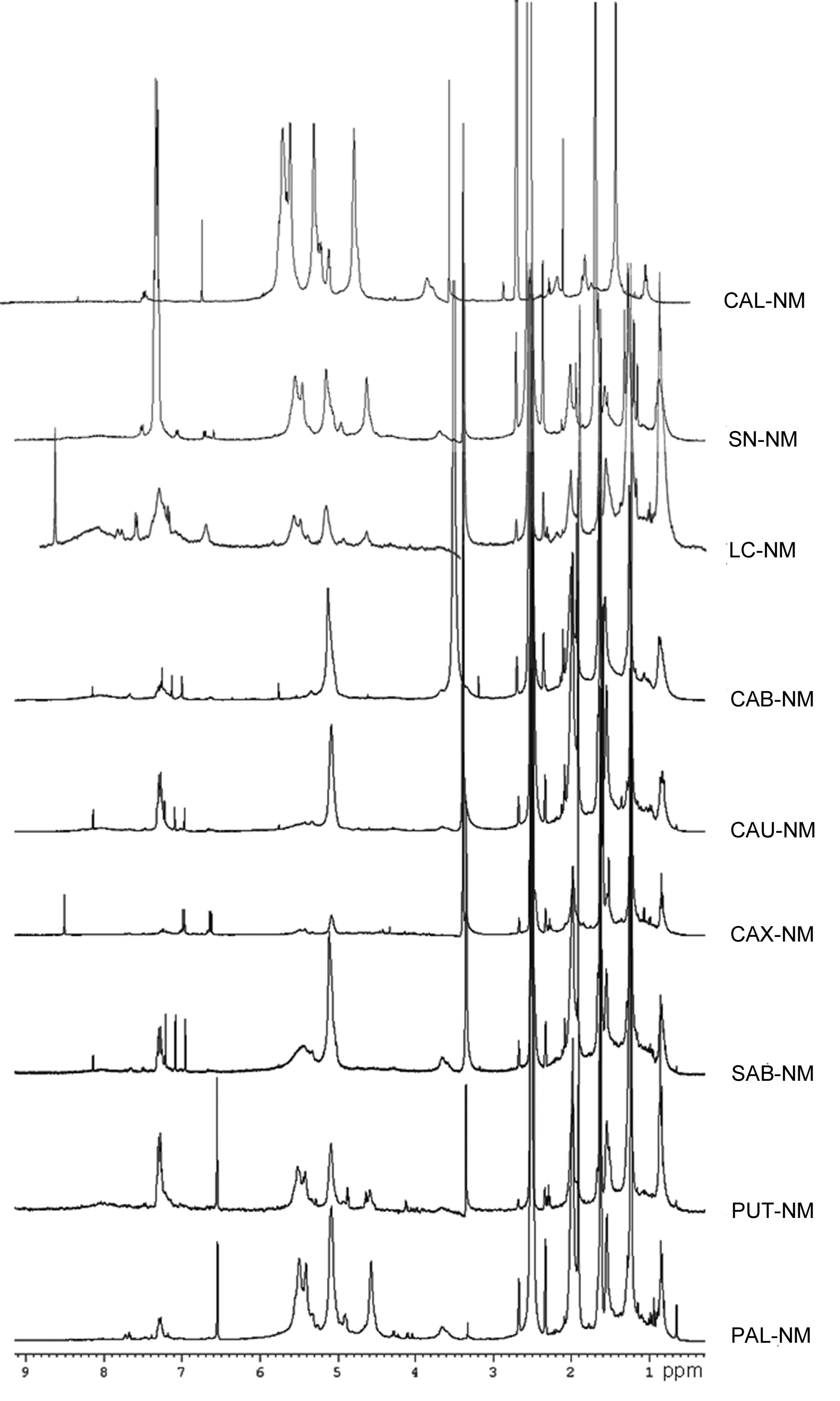

Supplement: Figure S3 — 1D proton NMR spectra of all NMs in DMSO-d6. (TIF) [file pone.0048490.s003.tif]

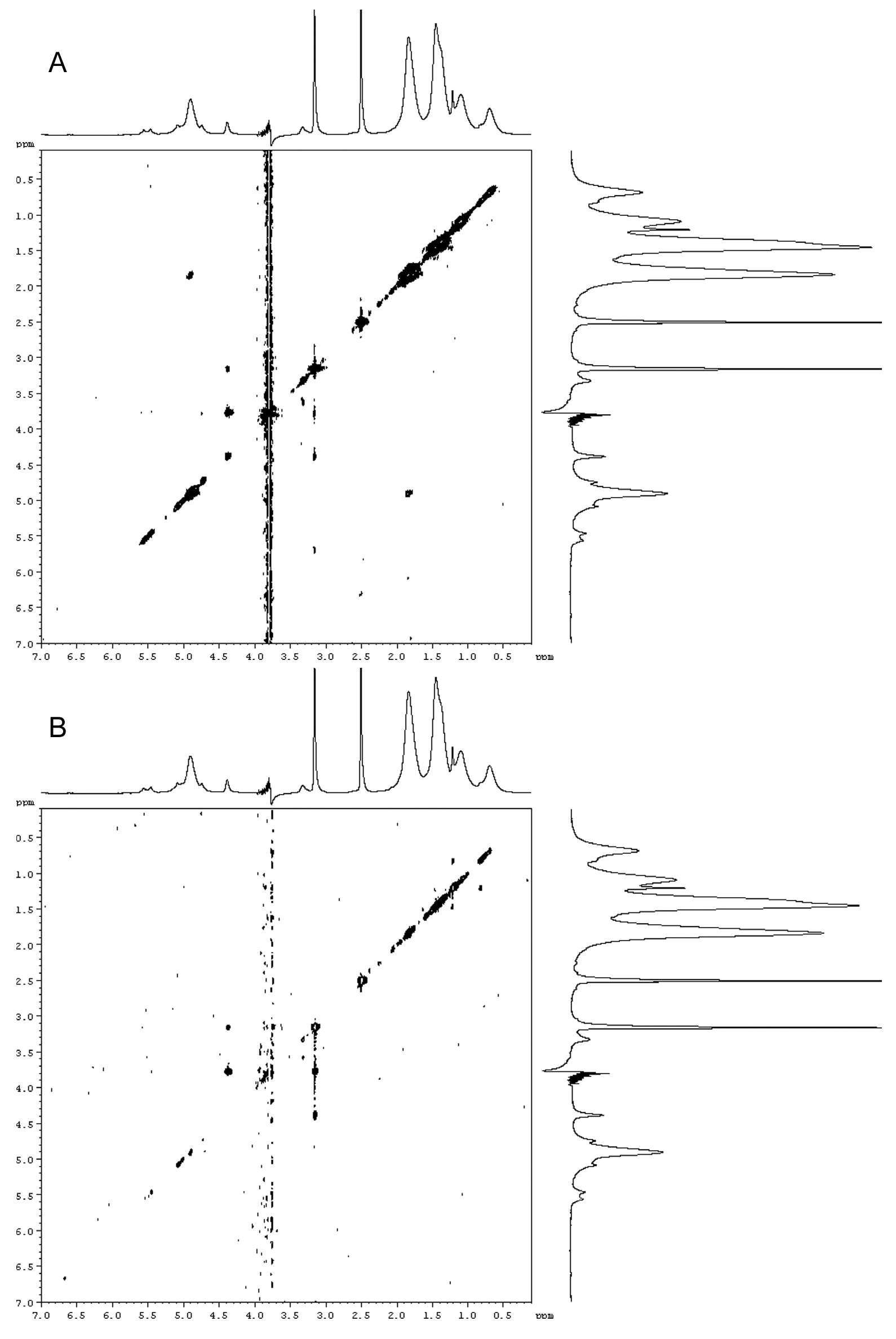

Supplement: Figure S4 — HR-MAS-TOCSY experiment of NM from PUT using different mixing times. (A) mixing time = 20 ms to show crosspeak between 4.9 and 1.8 ppm, and (B) mixing time = 60 ms to show crosspeaks 1.1–0.7 and 1.45–1.1 ppm. (TIF) [file pone.0048490.s004.tif]
