# Supplementary material for: Neuromelanins of Human Brain Have Soluble and Insoluble Components with Dolichols Attached to the Melanic Structure
Source: PLoS One. 2012 Nov 5;7(11):e48490. doi: 10.1371/journal.pone.0048490 (PMC3489676; doi:10.1371/journal.pone.0048490)
Supplement: Table S1 — Elemental analysis of NMs of the various brain areas. This table shows the striking similarities between PUT-NM, CAB-NM, CAX-NM, SAB-NM, PAL-NM and CAU-NM (data averaged as ‘other-NM’). (DOC) [file pone.0048490.s005.doc]

|  | SN-NM | PUT-NM | CAB-NM | CAX-NM | SAB-NM | PAL-NM | CAU-NM | CAL-NM | other-NM |
| --- | --- | --- | --- | --- | --- | --- | --- | --- | --- |
| % | (9) *^a^* | (5) | (6) | (3) | (2) | (2) | (2) | (2) | (20) |
| C | 52.81 | 59.12 | 60.93 | 62.16 | 59.27 | 59.74 | 58.97 | 52.17 | 60.03 |
| H | 6.47 | 7.67 | 7.92 | 8.13 | 8.06 | 7.90 | 7.84 | 7.12 | 7.92 |
| N | 7.33 | 5.72 | 5.49 | 6.10 | 5.86 | 5.21 | 5.17 | 3.42 | 5.59 |
| S | 2.93 | 2.95 | 2.93 | 2.87 | 2.61 | 2.79 | 3.31 | 1.43 | 2.91 |
| O | 24.62 | 18.84 | 19.71 | 17.91 | 19.59 | 19.99 | 19.77 | 31.19 | 19.30 |
| C/N | 7.2 | 10.3 | 11.1 | 10.2 | 10.1 | 11.5 | 11.4 | 15.3 | 10.7 |

**Table S1. Elemental analysis of NMs of the various brain areas.**

^a^ In parentheses the number of analyzed samples per area.

This table shows the striking similarities between PUT, CAB, CAX, SAB, PAL and CAU (data averaged as ‘other-NM’).
